# Novel Strategies for the Detection of Systolic and Diastolic Heart Failure

**DOI:** 10.2174/157340309788166651

**Published:** 2009-05

**Authors:** Cara Lodewijks-vd Bolt, Leo Baur, Jelle Stoffers, Timo Lenderink, Ron Winkens

**Affiliations:** 1 Dept. of Cardiology, Atrium Medical Centre Parkstad; 2 Care And Public Health Research Institute (CAPHRI), University Maastricht; 3 University of Maastricht, The Netherlands

**Keywords:** Heart failure, systolic dysfunction, diastolic dysfunction, brain natriuretic peptide, echocardiography.

## Abstract

The incidence and prevalence of dyspnea increases with age. Frequently, for the general practitioner with his limited diagnostic facilities, it is impossible to separate dyspnea from cardiac causes and non-cardiac causes. Without cardiac imaging it is also impossible to separate systolic dysfunction from diastolic dysfunction. After a thorough physical examination, initial screening of systolic and diastolic heart failure can be done by measurement of plasma NT-pro BNP or plasma BNP. Additionally a Chest X-Ray or ECG can be performed. To improve diagnostic performance an open access echocardiographic service can be initiated. Recent studies showed, that open access echocardiography can easily detect systolic and diastolic dysfunction in the community and can separate cardiac from non-cardiac dyspnea.

## EPIDEMIOLOGY

Heart Failure has become a main cardiac problem in the modern western society with a prevalence of 2-6% of the population or five million cases in the United States [1,2]. The calculated lifetime risk of developing heart failure in the Framingham Heart Study is about 20% [3]. Nearly half of the patients with heart failure have a preserved ejection fraction [4,5] . While death from coronary artery disease and stoke has been decreasing , there has been an increase in the prevalence, morbidity and mortality from congestive heart failure [6]. The prognosis of advanced heart failure is poor. Half of the patients with mild to moderate heart failure die within five years and half of the patients with severe heart failure die within two years [7]. Sudden death accounts for half of these deaths and is 6-9 times more common than in the general population. Also patients with preserved ejection fraction have a high all cause mortality: a 22 to 29% one year mortality and a 65% five year mortality [5]. Apart from this, heart failure is a common reason for hospitalisation and is a large expense for insurance companies. One third of the these costs are made in out-patient care and two thirds are spent in the hospital setting [8]. To prevent deterioration of cardiac function, it is important that systolic and diastolic dysfunction are diagnosed in an early stage. Unfortunately, heart failure is a complex clinical syndrome, which roughly can be grouped in predominant abnormalities of systolic function and in predominant abnormalities of diastolic function. Distiction between systolic dysfunction and diastolic dysfunction is complicated by the fact, that diastolic dysfunction nearly always coexists with systolic dysfunction. Therefore, heart failure is frequently misdiagnosed and the validity of the diagnosis in primary care is poor [9]. 

## DEFINITION OF DIASTOLIC HEART FAILURE

**Fig. (1):**
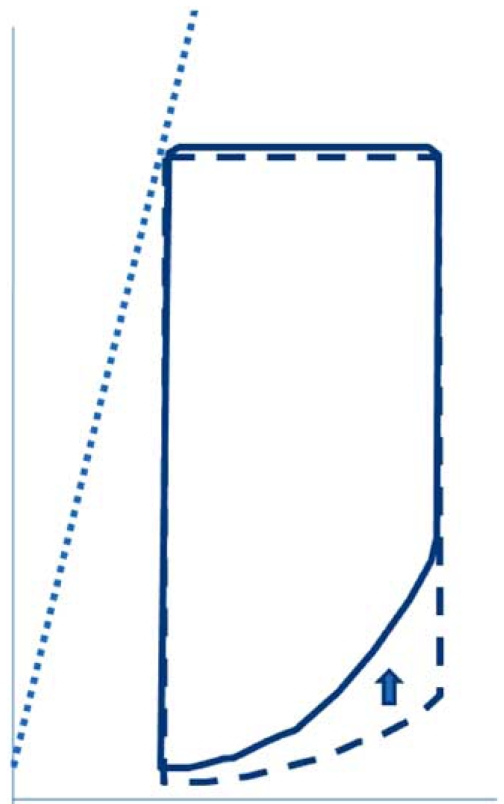
Diagram of diastolic left ventricular pressure-volume loop in a normal individual (interrupted line) and a patient with diastolic dysfunction (un-interrupted line) Note, that the end-diastolic and endsystolic volume is normal and that left ventricular ejection fraction is equal. However, the filling pressures in the patient with diastolic dysfunction is higher.

The term diastolic heart failure is used to describe group of patients with the clinical signs of congestive heart failure and normal diastolic volume and predominant abnormalities of in relaxation and increased passive stiffness [10,11]. There is slow and incomplete isovolumic pressure decline, slow and decreased early diastolic filling rate and volume and compensatory increased late diastolic filling by atrial contraction. In pure diastolic heart failure systolic left ventricular function is normal. The pathophysiological mechanism refers to the inability of the myofibrils to rapidly or completely return to their resting length. In this situation, the left ventricle cannot accept blood at low pressures and ventricular filling is slow or incomplete unless atrial pressure rises. With pressure-volume loops, diastolic dysfunction is characterized by increased left ventricular chamber stiffness (left and upward shift) of the diastolic pressure- volume relationship, which is the gold standard for determining diastolic heart failure (Fig. **1**).

## CLINICAL MANIFESTATION AND PHYSICAL EXAMINATION

The main approach to the patient with dyspnea is based on the history, physical examination, chest x-ray, and a series of laboratory diagnostic tests to establish the diagnosis, determine the etiology, and assess severity of heart failure. According to the Framingham data [12], heart failure can be diagnosed according to predefined clinical criteria: These criteria can be separated in major and minor clinical criteria. Major criteria are: Paroxysmal nocturnal dyspnea, orthopnea, elevated jugular venous pressure, pulmonary rales, third heart sound, cardiomegaly on chest x-ray, pulmonary edema on chest x-ray, weight loss 4.5 kg in five days in response to treatment of presumed heart failure. The minor criteria are: bilateral leg edema, nocturnal cough, dyspnea on ordinary exertion, hepatomegaly, pleural effusion, tachycardia (heart rate 120 beats/min), weight loss 4.5 kg in five days. The diagnosis of heart failure requires that 2 major or 1 major and 2 minor criteria cannot be attributed to another medical condition. Unfortunately, these symptoms are not specific and the clinical signs are not sensitive at all [13]. 

Severity of of symptomatic heart failure can be classified according to the criteria of the New York Heart Association, where: Class I indicate symptoms only at activity levels that would limit normal individuals Class II indicate symptoms with ordinary exertion Class III indicate symptoms with less than ordinary exertion and Class IV indicate at rest.

For a complete work-up of a heart failure patient, the initial evaluation includes the investigations in Table **1** [14].

## BRAIN NATRIURETIC PEPTIDES

Natriuretic peptides are released in respond to increased intracardiac volume and pressure [15] and are secreted in the ventricle. The plasma concentrations of of these pepides are frequently elevated in patients with heart failure and asymptomatic cardiac dysfunction [16,17]. Concentrations of Brain Natriuretic Peptide and NT-Brain Natriuretic Peptide rise with increasing dyspnea complaints [18]. Although BNP does better than other clinical variables to detect or to rule out heart failure in patients with dyspnea at the emergency department [19], BNP is less accurate in the detection of asymptomatic left ventricular dysfunction [20]. In this group of patients, BNP is a valuable tool to be rule out heart failure [21]. 

The level of BNP in plasma may also reflect diastolic dysfunction as has been shown in several studies [22,23]. Elevated plasma levels of BNP can help to reinforce the diagnosis of diastolic dysfuncton in patients with a normal systolic function determined with any imaging technique. On the other hand can a low BNP level rule out diastolic dysfunction. One has to keep in mind, that BNP is not a stand alone diagnostic test and must be interpreted in a wider clinical context. Small increases are not specific for left ventricular dysfunction, but can also be observed in patients with right ventricular dysfunction and renal insufficiency [24,25]. Additionally one has to consider that the normal range of BNP is specific to age, sex and the used assay [26].

## DIASTOLIC DYSFUNCTION

Diastolic heart failure is very common, especially in elderly women [27]. Patients with diastolic heart failure often have left venricular hypertrophy and relatively well preserved systolic function. Diastolic dysfunction is now more readily recognized and may account for more than half of the hospital admisions for heart failure [28]. Treatment of diastolic heart failure differs from the treatment of systolic heart failure [29]. 

## ECHO-DOPPLER IMAGING

Diastolic function can be measured with echo-Doppler imaging by interrogation of mitral inflow and pulmonary vein flow using pulsed-wave Doppler and tissue Doppler imaging of mitral annular motion. Using pulsed-wave Doppler at the mitral valve annulus, several parameters can be measured . Left ventricular *isovolumic relaxation time* (IVRT) is the time between aortic valve closure and mitral valve opening and represents dynamic left ventricular relaxation [30]. Normal values are 65 ± 20 milliseconds [31]. The IVRT is prolonged (> 100 ms) in patients with diastolic dysfunction and normal filling pressures. IVRT is shortened if mitral valve opening occurs early, which can be seen in young adults and patients with increased mean left atrial pressure due to restrictive cardiomyopathy [32]. 

Pulsed Doppler interrogation of the mitral annular flow reveals a peak E wave and a peak A wave. The peak E wave measures the early diastolic transmitral pressure gradient and thus reflects the rapid filling phase. Normal values are between 70 and 100 cm/s. The peak A wave measures the late diastolic transmitral pressure gradient and thus represents atrial contraction. The normal value is 45 to 70 cm/s. The overall characterization of left ventricular filling is by the *E/A ratio*, which normally is less than one See also Fig. (**2**).

*Mitral deceleration time* reflects left ventricular compliance in early diastole and is normally between 160 and 220 msec. In normal young adults, there is a predominance of early diastolic filling (E wave) with only 10% to 15% filling caused by atrial contraction. With the normal aging process, relaxation slows and causes reduced filling in early diastole and increased filling by atrial contraction [33]. In patients with cardiac relaxation abnormalities, three abnormal filling patterns can be recognized. These filling patterns are known as *impaired relaxation* , *pseudonormalization* , and *restrictive filling* [34]. With a slight elevation of left atrial pressure from 8 to 14 mm Hg, the E wave decreases and the A wave increases, resulting in an E/A ratio less than 1. The deceleration time is more than 240 msec. 

A more severe compliance reduction and increase of left atrial pressure to 15 to 22 mm Hg results in a left ventricular filling pattern that appears normal but actually is not; this pattern is called a *pseudonormal filling pattern*. With this pattern, the E/A ratio is 1 to 1.5, and the deceleration time is normal. In more advanced disease, high atrial pressures (> 22 mm Hg) cause blood to rapidly fill the slowly relaxing left ventricle in early diastole. A marked increase in early left ventricular diastolic pressure results in only limited additional filling. The pulsed wave (PW) flow pattern is now known to be restrictive with a high velocity of the E wave, an E/A ratio more than 2, and a short deceleration time of less than 160 msec.

Because transmitral flow patterns are load-sensitive, additional measurement of pulmonary vein flow is helpful is assessing left ventricular filling [35]. With experience, high-quality PW Doppler transthoracic recordings can be obtained in 90% of patients. 

The normal pulmonary vein flow shows a systolic flow (S) that is higher than the diastolic flow (D) with an atrial reversal flow (A).

Patients with impaired relaxation have a normal pulmonary vein flow pattern. Patients with pseudonormalization and restrictive filling have a decrease of the S wave compared with the D wave and a pulmonary vein A wave flow reversal of > 35 cm/s. 

### Tissue Doppler Imaging (TDI) 

TDI is a new modality that measures the velocity of the myocardium during the cardiac cycle (i.e., diastolic function) instead of blood flow [35]. TDI velocities can be displayed as spectral Doppler signals, color-encoded M-mode images**,** and two-dimensional color maps. Imaging is usually performed from the apical window. From this view, the axial motion of the ventricle is parallel to the transducer axis, and velocities are primarily related to left ventricular contraction and relaxation. The pulsed-wave sample volume is mostly placed within the septal and lateral regions of the mitral annulus.

Normally, a systolic part (Vs), an early diastolic part (Ve or E’), and a late diastolic part (Va or A’) can be differentiated. If diastolic function is normal, the peak of Ve tends to be earlier than the peak of the mitral flow E wave. Patients with impaired ventricular relaxation have a Ve/Va ratio less than 1 [36]. Tissue Doppler imaging seems to be less preload-dependent than mitral flow Doppler. The most robust index of diastolic function is the combination of pulsed wave Doppler of the mitral inflow in combination with tissue Doppler imaging. The ratio of early peak transmitral inflow velocity (E) to the tissue Doppler velocity of early diastolic myocardial ascent (E') is a reliable guide to elevated pulmonary capillary wedge pressure [37]. An E/E’ > 12 predicts an elevated wedge pressure, while a ratio < 8 predicts a normal wedge pressure.

## SYSTOLIC DYSFUNCTION

Patients with systolic dysfunction present with left ventricular dilation and a reduced ejection fraction. Left ventricular damage from any cause can initiate and perpetuate a process called left ventricular remodeling. Ultimately this process culminates in an increase of left ventricular enddiastolic and endsystolic volumes, a reduced ejection fraction and an increase of left ventricular wall stress [38,39]. Heart failure is accompanied by changes in the cardiac myocyte and the interstitium [40]. 

Patients, who have dilated hearts and a reduced left ventricular ejection fraction may occasionally be asymptomatic and can be identified by echocardiographic population screening or screening of plasma brain natriuretic peptide [41,42]. 

**Fig. (3):**
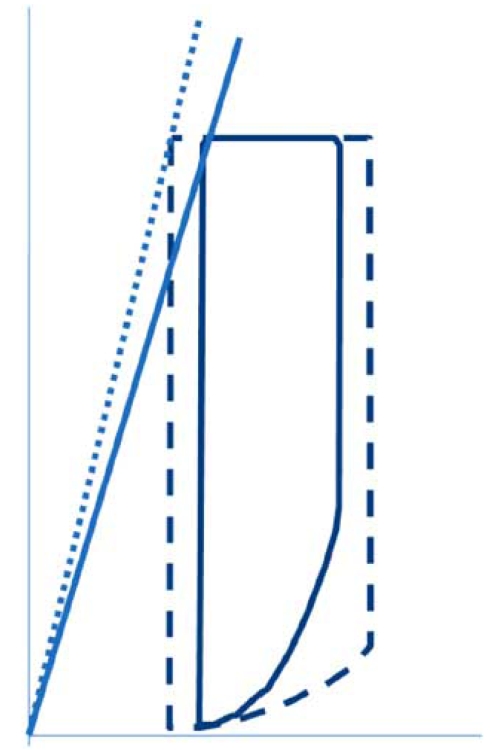
Pressure-volume loops in a patient a normal individual (interrupted line) and a patient with systolic dysfunction (un-interrupted line). Systolic dysfunction is characterised by decreased stoke work (the area inside the pressure-volume loop) and decreased ejection fraction. The slope of the endsystolic pressure-volume relation is also decreased and moved downward and to the right [10].

Measurement with pressure-volume loops is the gold standard for assessment of left ventricular systolic dysfunction (Fig. **3**).

In clinical practice, echocardiography is most frequently used technique for assessement of left ventricular systolic dysfunction. 

With echocardiography, left ventricular systolic function can be assessed in several ways. 

An echocardiographic examination can start with an M-Mode with measurement of left ventricular internal dimensions in diastole and in systole. From these parameters fractional shortening and velocity of circumferential shortening can be calculated. An indirect of systolic dysfunction is the EPSS or E-point septal separation. Normally, the mitral valve E point is within 6 mm of the left side of the ventricular septum. In the presence of a decreased ejection fraction, this distance is increased. A patient with marked left ventricular dysfunction with an EPSS of 2.4 cm. The left ventricle is dilated and has a decreased fractional shortening.

Two dimensional echocardiography provides inherently superior spatial resolution for determining left ventricular size and function. Several methods have been developed for measurement of left ventricular volume and left ventricular ejection fraction. The most common method for determining ventricular volumes is the Simpson rule or the “rule of disks”. It requires recording of an apical four- or two chamber view from which the endocardial border is outlined in end-diastole and end-systole. The ventricle is then mathematically along its long axis into a series of disks of equal height. The individual disk volume is calculated as height x disk area, where height is assumed to be the total length of the left ventricular long axis divided by the number of segments or disks [43]. 

A more accurate technique to determine ventricular volume, especially in abnormally shaped ventricles, is with three dimensional echocardiography. The major technical limitation of three dimensional volume determination has been the cumbersome nature of reconstruction techniques [44]. 

**Fig. (4):**
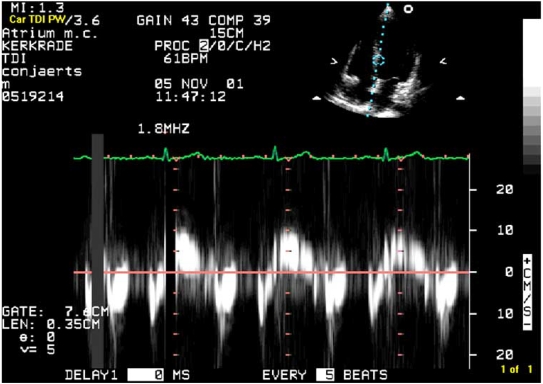
Pulsed wave Doppler at the basal septum (Fig. **2**). The first positive wave is the systolic wave (Vs) the first negative wave, the early diastolic wave (Ve) and the second negative wave the late diastolic wave (Va).

Tissue Doppler Myocardial Imaging can be applied for the measurement of longitudinal systolic left ventricular wall motion. (Fig. **4**). In patients with dilated cardiomyopathy, mitral annular systolic velocities are significantly lower compared to normals and correlate well with left ventricular ejection fraction [45]. Next to this Tissue Doppler Imaging is frequently used to quantify left ventricular asynchrony and the effects of biventricular pacing [46].

Regional left ventricular function can be calculated with the myocardial velocity gradient or by strain rate imaging [47]. Strain is a change in length corrected for the original length or the fractional or percentage change from the original or unstressed dimension and reflects deformation of a structure. Strain rate is the rate of this deformation and is a strong index of left ventricular contractility. 

## OPEN ACCESS ECHOCARDIOGRAPHY

As previously oulined, systolic or diastolic left ventricular dysfunction is difficult to identify solely on the basis of signs and symptoms [48]. The most easy may to have a correct diagnosis is by echocardiography. The last decade several projects in and outside the UK have been developed to make echocardiography directly available to general practitioners [27,49-52]. Open access echocardiography is defined as echocardiography requested by a general practitioner without prior clinical assessment by a cardiologist [53].

The service has been very succesful in detecting left or right ventricular dysfunction and the etiology of cardiac murmurs. The echocardiogram frequently gives the general practitioner additional information with minor discomfort for the patient. The costs of setting up open access services can be considerable. Therefore there have been concerns about this service [53]. However, there have been no formal evaluations of the cost effectiveness of this service. Another possible disadvantage of an open access service could be overload of the department of echocardiography if strict criteria for referral are not taken. Unneccecary referrals can be avoided by take in account the rules as they have been described by the Britisch Working Group on open access echocardiography [52].

Until now the service is has gained only acceptance in the United Kingdom and the Netherlands. The service is cost-effective and can avoid unneccesary referrals to the outpatient clinic of cardiology.

Another way to limit the number of referrals is by prescreening of patients by making electrocardiograms or by measurement of plasma BNP [54]. Because a normal electrocardiogram and a normal BNP excludes systolic or diastolic dysfunction, this process can limit the number of unneccessary referrals.

## Figures and Tables

**Fig. (2):**
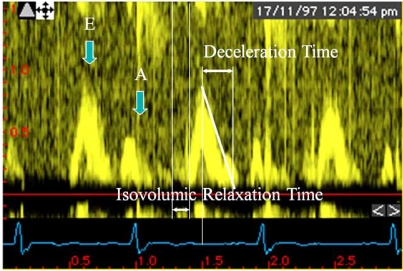
Pulsed-wave doppler mitral flow velocity pattern showing different variables representative of diastolic function: peak E-wave velocity (E); Peak A-wave velocity (A); Ratio between E and A wave velocities; isovolumic relaxation time.

**Table 1:** ACC/AHA Guideline Summary: Initial Evaluation of Patients with Heart Failure

*Class I: There is evidence and/or general agreement that the initial evaluation of patients presenting with HF should include the following:*
A complete history and physical examination to identify cardiac and noncardiac disorders or behaviors that might cause or accelerate the development or progression of HF. A careful history of current and past use of alcohol, illicit drugs, standard or "alternative" therapies, and chemotherapy drugs. An assessment of the ability to perform routine and desired activities of daily living. An assessment of the volume status, orthostatic blood pressure changes, height and weight, and calculation of body mass index. Laboratory studies including complete blood count, urinalysis, serum electrolytes (including calcium and magnesium), blood urea nitrogen, serum creatinine, fasting blood glucose (glycohemoglobin), lipid profile, liver function tests, and serum thyroid-stimulating hormone. A twelve-lead electrocardiogram and chest radiograph (posteroanterior and lateral). Two-dimensional echocardiography with Doppler to assess left ventricular ejection fraction (LVEF), left ventricular size, wall thickness, and valve function. Radionuclide ventriculography can be performed to assess LVEF and volumes. Coronary arteriography if there is a history or angina or significant ischemia unless the patient is not eligible for revascularization of any kind.
*Class IIa: The weight of evidence or opinion is in favor of benefit from performing the following studies as part of the initial evaluation of patients presenting with HF:*
Coronary arteriography in patients who have chest pain that may or may not be of cardiac origin who have not had a prior evaluation of their coronary anatomy and are eligible for coronary revascularization. Coronary arteriography in patients with known or suspected coronary artery disease who do not have angina and are eligible for revascularization. Noninvasive imaging to detect myocardial ischemia and viability in patients with known or suspected coronary artery who do not have angina and are eligible for revascularization. When the contribution of HF to exercise limitation is uncertain, maximal exercise testing with or without measurement of respiratory gas exchange and/or blood oxygen saturation. To identify candidates for cardiac transplantation or other advanced treatments, maximal exercise testing with measurement of respiratory gas exchange. In selected patients, screening for hemochromatosis, sleep disturbed breathing, or human immunodeficiency virus (HIV) infection. When suspected clinically, diagnostic tests for rheumatologic disease, amyloidosis, or pheochromocytoma. Endomyocardial biopsy when a specific diagnosis is suspected that would influence therapy. Measurement of serum B-type natriuretic peptide (BNP) in the urgent care setting if the clinical diagnosis of HF is uncertain.
*Class IIb: The weight of evidence or opinion is less well established for the following testing in the initial evaluation of patients with HF:*
Noninvasive imaging to define the likelihood of coronary artery disease in patients with left ventricular dysfunction Holter monitoring in patients who have a history of myocardial infarction and are being considered for electrophysiologic study to document the inducibility of ventricular tachycardia.
*Class III: There is evidence and/or general agreement that the following tests are not useful or may be harmful in the initial evaluation of patients with HF:*
Routine endomyocardial biopsy in the absence of suspicion of a specific diagnosis that would influence therapy suspected. Routine signal-averaged electrocardiography. Routine measurement of serum neurohormones other than BNP (eg, norepinephrine or endothelin).

**Table 2:** Open Access Echocardiograhy is Indicated in Patients with Dyspnea or Peripheral Edema if:

Clinical assessment, ECG and other tests (BNP, Chest X-Ray) cannot eliminate cardiac disease as a cause.
**Cardiology referral should be considered if:**
Dyspnea and/or peripheral edema is accompanied by unequivocal signs of cardiac disease, or major ECG abnormalities, or major abnormalities of a chest X-Ray or an abnormal plasma BNP.
**Referral is not indicated in patients with dyspnea or peripheral edema, if:**
There are complete normal findings on cardiovascular assessment, a normal ECG, a normal plasma BNP or an alternative explanation for the signs and symptoms.
